# Development of Polyelectrolyte Complexes for the Delivery of Peptide-Based Subunit Vaccines against Group A *Streptococcus*

**DOI:** 10.3390/nano10050823

**Published:** 2020-04-26

**Authors:** Lili Zhao, Wanli Jin, Jazmina Gonzalez Cruz, Nirmal Marasini, Zeinab G. Khalil, Robert J. Capon, Waleed M. Hussein, Mariusz Skwarczynski, Istvan Toth

**Affiliations:** 1 School of Chemistry & Molecular Biosciences, The University of Queensland, St. Lucia, QLD 4072, Australia; lili.zhao@uq.net.au (L.Z.); wanli.jin@uq.net.au (W.J.); nirmal.marasini@uq.net.au (N.M.); w.hussein@uq.edu.au (W.M.H.); 2 Diamantina Institute, Translational Research Institute, The University of Queensland, Wooloongabba, QLD 4102, Australia; j.gonzalezcruz@uq.edu.au; 3 Institute for Molecular Bioscience, The University of Queensland, St. Lucia, QLD 4072, Australia; z.khalil@imb.uq.edu.au (Z.G.K.); r.capon@imb.uq.edu.au (R.J.C.); 4 Pharmaceutical Organic Chemistry Department, Faculty of Pharmacy, Helwan University, Helwan 11795, Egypt; 5 School of Pharmacy, The University of Queensland, Woolloongabba, QLD 4102, Australia

**Keywords:** lipopeptide subunit vaccine, liposomes, polyelectrolyte complexes, nanoparticles, group A *streptococcus*

## Abstract

Peptide subunit vaccines hold great potential compared to traditional vaccines. However, peptides alone are poorly immunogenic. Therefore, it is of great importance that a vaccine delivery platform and/or adjuvant that enhances the immunogenicity of peptide antigens is developed. Here, we report the development of two different systems for the delivery of lipopeptide subunit vaccine (LCP-1) against group A streptococcus: polymer-coated liposomes and polyelectrolyte complexes (PECs). First, LCP-1-loaded and alginate/trimethyl chitosan (TMC)-coated liposomes (Lip-1) and LCP-1/alginate/TMC PECs (PEC-1) were examined for their ability to trigger required immune responses in outbred Swiss mice; PEC-1 induced stronger humoral immune responses than Lip-1. To further assess the adjuvanting effect of anionic polymers in PECs, a series of PECs (PEC-1 to PEC-5) were prepared by mixing LCP-1 with different anionic polymers, namely alginate, chondroitin sulfate, dextran, hyaluronic acid, and heparin, then coated with TMC. All produced PECs had similar particle sizes (around 200 nm) and surface charges (around + 30 mV). Notably, PEC-5, which contained heparin, induced higher antigen-specific systemic IgG and mucosal IgA titers than all other PECs. PEC systems, especially when containing heparin and TMC, could function as a promising platform for peptide-based subunit vaccine delivery for intranasal administration.

## 1. Introduction

Vaccination is perceived as one of the most effective approaches for the prevention and containment of infectious diseases. Vaccines are developed to trigger immunity against specific pathogens once administered into the body. Traditional vaccines are normally produced from attenuated or inactivated forms of whole pathogenic microorganisms. However, the use of whole pathogens is associated with potential drawbacks, such as the risk of infection, auto-immune and strong allergic responses, low yield of production, and low storage stability [1]. In order to avoid these disadvantages, subunit vaccines, such as peptide vaccines, have emerged [2,3]. Peptide-based subunit vaccines contain the most essential fragments of a pathogen needed to stimulate desired immune responses. However, without the assistance of an adjuvant, peptides cannot stimulate long-lasting immune responses [4].

In addition to widely used adjuvants, such as aluminum salts, oil-in-water emulsions, and lipid-based adjuvants [5], immunostimulatory delivery systems have been adopted in the development of subunit vaccines [6,7,8]. These systems can protect antigens from degradation and stimulate desired immune responses through various pathways, including (a) the depot effect to endow a long-lasting release of loaded antigens [5], (b) incorporation of target moieties to facilitate antigen recognition and uptake by antigen presenting cells (APCs) [9,10], and (c) muco-adhesive and mucus-penetrating properties to enhance mucosa residence time of antigens [11,12]. Various vaccine delivery systems have been developed, such as liposomes, poly(lactic-co-glycolic acid) nanoparticles, emulsions, and chitosan-based polymeric nanoparticles [13,14,15,16]. These delivery systems can mimic pathogens’ surface features, like size, charge, and shape, in order to be readily recognized by the immune system [17].

Liposomes are vesicles comprised of lipid bilayers that are formed from naturally derived, biodegradable, and nontoxic phospholipids [18,19]. They have been extensively studied for vaccine delivery and some have reached clinical applications [20]. Liposomes’ inherent adjuvanticity has long been confirmed [15]. Liposomes composed of dipalmitoylphosphatidylcholine (DPPC), dimethyldioctadecylammonium bromide (DDAB), and cholesterol were fabricated to intranasally deliver subunit vaccines against group A streptococcus (GAS), and generated potent humoral immune responses in both outbred and inbred mice [21,22]. Surface coating for liposomes has been adopted to address their muco-adhesive properties and antigen instability in the gastrointestinal tract [23]. Polymer-coated liposomes promoted the production of antigen-specific systemic IgG and mucosal IgA antibodies following oral immunization in outbred mice.

Polyelectrolyte complexes (PECs), also known as nanocomplexes, are nanoparticles produced based on electrostatic interactions between oppositely charged polyelectrolytes [24,25]. Polysaccharides, such as chitosan, heparin, chondroitin sulfate, and sodium alginate, are typical representatives of the biodegradable and biocompatible polyelectrolytes, and have been massively investigated and employed in drug delivery systems [26,27]. PECs composed of polysaccharides have also shown great promise for vaccine delivery because of their adjuvating potential. Chitosan and its derivatives, for instance, trimethyl chitosan (TMC), have been reported as potent adjuvants for mucosal vaccines owing to their muco-adhesivity, positive charge, and binding affinity with β-glucan carbohydrate receptors (Dectin-1 and mannose receptors) presented on APCs [28,29]. Dextran can be recognized by mannose receptors, so can also promote antigen uptake by APCs [30], while hyaluronic acid can facilitate T-cell activation by binding with CD44 [31]. Chondroitin sulfate promotes an antigen-specific Th1 immune response; however, the mechanism through which this happens is not clear [24,32,33]. PEC nanoparticles composed of GAS-derived peptide antigen, dextran, and TMC were efficiently taken up by dendritic cells (DCs) and induced DC maturation, which successfully initiated systemic and mucosal immune responses in outbred mice [34]. Alginate has also been used extensively as a delivery material in vaccine formulations [35,36].

**Figure 1:**
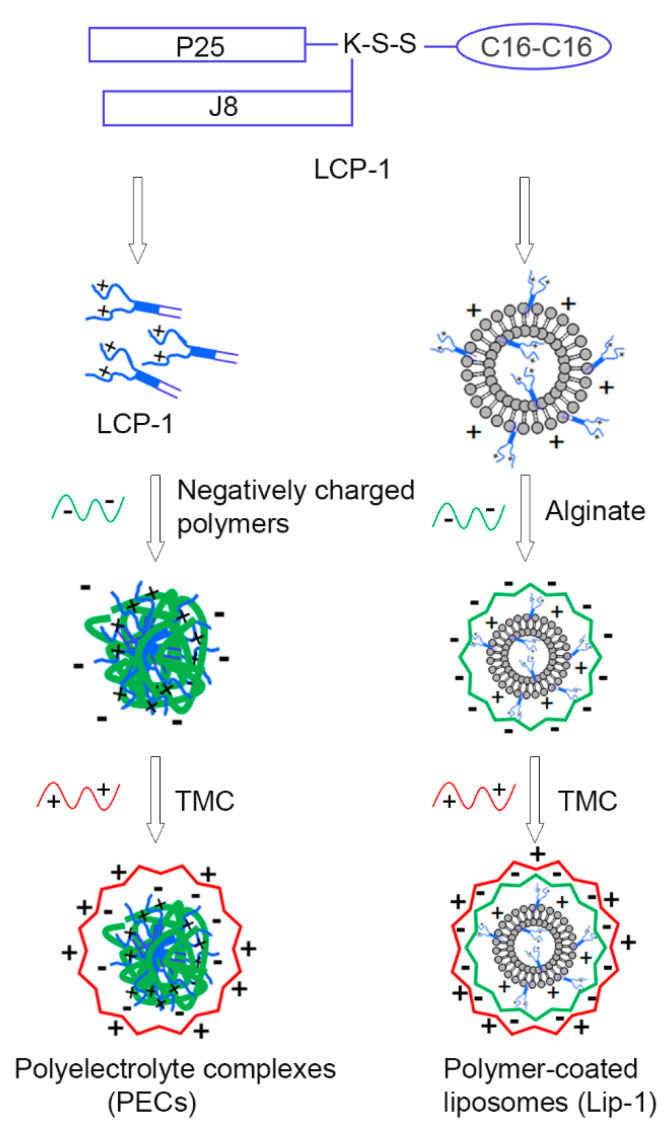
Schematic illustration of delivery systems designed for LCP-1: polyelectrolyte complexes (PECs) and polymer-coated liposomes. PECs were produced by mixing LCP-1 with negatively charged polymer, then coated with positively charged trimethyl chitosan (TMC); while polymer-coated liposomes were produced by encapsulating LCP-1 into liposomes, followed by coating with alginate and TMC. C16: lipid moiety 2-amino-d,l-hexadecanoic acid, J8: B cell epitope derived from GAS M protein (QAEDKVKQSREAKKQVEKALKQLEDKVQ), P25: universal T helper epitope (KLIPNASLIENCTKAEL), K: lysine, S: serine.

The incorporation of lipid moieties into peptide epitopes is another adjuvanting strategy that works by increasing antigens’ immunogenicity through targeting peptides to Toll-like receptors 2 (TLR-2) on DCs [37,38]. A self-adjuvanting lipid core peptide (LCP) system was designed that incorporated peptide epitopes, a lipid moiety and a linker/carrier in one molecular entity [39]. To date, LCP systems have been used in the delivery of vaccines against a variety of pathogens, including group A *streptococcus* (GAS), hookworm, human papilloma virus, and malaria [40,41]. GAS is a Gram-positive bacterium that typically invades the human throat and skin. GAS can cause multiple diseases, such as strep throat, post-streptococcal glomerulonephritis, rheumatic heart disease, and rheumatic fever [42,43]. There is an urgent need for an effective, affordable, and safe vaccine for GAS. We developed an LCP-based vaccine candidate against GAS (LCP-1), comprising B cell epitope J8 (QAEDKVKQSREAKKQVEKALKQLEDKVQ) derived from the M protein of GAS, universal T-helper epitope P25 (KLIPNASLIENCTKAEL), and lipid moiety 2-amino-d,l-hexadecanoic acid (C16) (Figure 1).

Herein, we developed a series of polysaccharide-based nanoparticles to further improve LCP-1′s efficiency in inducing adaptive immune responses following intranasal delivery. First, LCP-1-loaded and alginate/TMC-coated liposomes (Lip-1) and LCP-1/alginate/TMC PEC nanoparticles (PEC-1) were constructed and examined for their ability to elicit humoral immune responses in outbred Swiss mice. Based on this pilot study, the PEC system was chosen for further development of intranasal vaccine candidates against GAS. In PECs systems, cationic TMC has been reported as a potent adjuvant/delivery system for mucosal vaccines [34,44,45,46]; however, the role of negative polymers has not been tested. Therefore, we examined the influence of anionic polymers in PEC delivery systems on LCP-1 vaccine activity against GAS. A series of PECs were prepared by mixing LCP-1 with anionic polymers (including alginate, chondroitin sulfate, dextran, hyaluronic acid, and heparin), then coated with TMC (Figure 1). The efficacy of these PECs to stimulate the production of systemic and mucosal antibodies was evaluated in outbred mice.

## 2. Materials and Methods

### 2.1. Materials

Protected l-amino acids, rink amide *p*-methylbenzhydrylamine (MBHA) resin, and 1-[bis(dimethylamino)methylene]-1H-1,2,3-triazolo [4,5-b]pyridinium 3-oxid hexafluoro- phosphate (HATU) were purchased from Novabiochem (Hohenbrunn, Germany) and Mimotopes (Melbourne, Australia). Peptide synthesis-grade dichloromethane (DCM), *N,N*-diisopropylethylamine (DIPEA), and trifluoroacetic acid (TFA) were purchased from Merck (Hohenbrunn, Germany). Chitosan (low molecular weight, 75–85% deacetylated), sodium alginate (low viscosity), dextran sodium, chondroitin sulfate, heparin sodium, cholera toxin B subunit (CTB), horseradish peroxidase (HRP)-conjugated goat anti-mouse IgG and IgA, high performance liquid chromatography (HPLC) solvent (acetonitrile), and all other reagents were purchased from Sigma-Aldrich (Sydney, Australia). Dipalmitoylphosphatidylcholine (DPPC), cholesterol, and dimethyldioctadecylammonium bromide (DDAB) were bought from Avantis Polar, Inc. (Sydney, Australia). RMPI medium was obtained from Gibco^®^ Life Technologies (Carlsbad, CA, USA). Bovine serum albumin, PE-Cy7 antimouse CD11c, BV421 antimouse MHC-II, APC antimouse CD86, and PE antimouse CD80 were purchased from eBioscience (San Diego, CA, USA). Monophosphoryl Lipid A (MPLA) was purchased from InvivoGen (San Diego, CA, USA). Hyaluronic acid was obtained from Shandong Freda Biotechnology Co. Ltd. (Shandong, China) as a gift. Trimethyl chitosan was synthesized as reported [23].

### 2.2. Methods

#### 2.2.1. Synthesis of the Lipopeptide Vaccine Candidate

Lipopeptide vaccine candidate LCP-1 was synthesized on *p*-MBHA rink-amide resin (substitution degree: 0.59 mmol/g) using standard microwave-assisted solid phase peptide synthesis, as previously reported [44]. Purification of the obtained crude compound was performed via HPLC (C4 column) [Yield: 25.7%]. Purified LCP-1 was measured by analytical HPLC and electrospray ionization mass spectrometry (ESI-MS). HPLC was performed at a flow rate of 1 mL/min and ultraviolet (UV) detection at a wavelength of 214 nm on a Vydac analytical C4 column (5 µm, 4.6 mm × 250 mm). Retention time t_R_ = 23.0 min. MW = 6014.2 g/mol. ESI-MS: m/z 1203.8 (calculated 1203.8) [M + 5H]^5+^; 1003.1 (calculated 1003.4) [M + 6H]^6+^; 860.0 (calculated 860.2) [M + 7H]^7+^; 752.8 (calculated 752.8) [M + 8H]^8+^; 669.2 (calculated 669.2) [M + 9H]^9+^ (See Supplementary Materials Figures S1 and S2).

#### 2.2.2. Preparation of Polymer-Coated Liposomes

LCP-1-loaded liposomes were prepared according to the thin film hydration method [23]. The lipids (DPPC:DDAB:cholesterol = 4 mg:0.2 mg:1 mg) were collectively dissolved in 0.6 mL chloroform, then mixed with LCP-1 (1 mg) dissolved in 1 mL methanol in a round-bottomed flask. Lipid film formed on the wall of the flask by slow evaporation of the organic solvent under reduced pressure using a rotary evaporator. The residue solvent was removed by freeze-drying overnight. The lipid film was then rehydrated with 1 mL Milli-Q water. The obtained liposomes were extruded through a 200 nm filter membrane using an Avanti^®^ Mini Extruder to achieve monolamellar liposomes with uniform size. The liposomes were then successively surface coated with alginate and TMC to produce liposome (LCP-1)/alginate/TMC (e.g., Lip-1). Separate stock solutions of alginate and TMC were prepared at concentrations of 2 mg/mL in Milli-Q water. The alginate stock solution was diluted into a series of portions to concentrations of 0.1, 0.2, 0.4, 0.6, 0.8, and 1 mg/mL. Similarly, the TMC stock solution was diluted to 0.2, 0.4, 0.6, 0.8, 1, and 1.2 mg/mL. Aliquots (50 µL) of each concentration of the alginate solution (containing 10, 20, 40, 60, 80, and 100 µg of alginate) were added dropwise to liposome solutions (containing 100 µg LCP-1) and gently stirred for 1 h at room temperature. The particle size and surface charge were monitored throughout the process by dynamic light scattering (DLS). The amount of TMC required for coating liposomes/alginate particles was screened and selected likewise. Aliquots (50 µL) of each concentration of the TMC solution (containing 20, 40, 60, 80, 100, and 120 µg of TMC) were added dropwise to liposome/alginate solutions (containing 100 µg LCP-1) and gently stirred for 1 h at room temperature and followed by DLS analysis.

#### 2.2.3. Preparation of Polyelectrolyte Complexes (PECs)

PECs were prepared via titration [47,48]. Stock solutions of polymers, including alginate, chondroitin sulfate, dextran, hyaluronic acid, heparin, and TMC, were prepared at concentrations of 2 mg/mL in Milli-Q water. LCP-1 (1 mg) was dissolved in 1 mL Milli-Q water. To optimize PEC-1 (LCP-1/alginate/TMC) preparation, 50 µL of each of the alginate solutions (containing 5, 10, 20, 30, 40, and 50 µg) was added into LCP-1 solution (100 µg) dropwise and the mixtures were sonicated for 4 min (2 min × 2 times) using a probe-type sonicator (Ultrasonic Homogenizer Model 3000, Biologics, Inc.: Cary, NC, USA) at 120 W in an ice bath. The mixtures were then stirred continuously for 1 h at room temperature to stabilize the complexes. Afterwards, 50 µL of TMC solution (equivalent to 20, 30, 50, 60, 80, and 100 µg of TMC) was slowly added into the LCP-1/alginate complexes, then stirred for 1 h to produce PEC-1 (LCP-1/alginate/TMC). PEC-2 (LCP-1/chondroitin sulfate/TMC), PEC-3 (LCP-1/dextran/TMC), PEC-4 (LCP-1/hyaluronic acid/TMC) and PEC-5 (LCP-1/heparin/TMC) were prepared following the same procedure.

#### 2.2.4. Morphology

The shape and surface morphology of LCP-1-loaded nanoparticles were examined through transmission electron microscopy (Hitachi HT7700, Hitachi: Tokyo, Japan). Nanoparticles (PEC-1 to PEC-5, 0.5 mg/mL) were dropped onto carbon-coated copper grids, then stained with 0.5% (*w*/*v*) phosphotungstic acid for 30 s. They were then air dried at room temperature.

#### 2.2.5. Particle Size and Zeta Potential

The particle size, polydispersity index (PDI), and zeta potential of Lip-1 and PEC-1–PEC-5 were measured by DLS at a back-scattering angle of 173 °C using a Malvern Zetasizer Nano-ZS (Malvern Instruments Ltd.: Malvern, UK). Each measurement was performed five times.

#### 2.2.6. Cytotoxicity Study

Cytotoxicity assessment of the nanoparticles was performed using SW620 (human colorectal cancer cell line), NCIH-460 (human lung cancer cell line), and HEK-293 cells (human kidney cell line). Cells were seeded into 96-well plates at a density of 2 × 10^3^ cells/well for SW620 and 5 × 10^3^ cells/well for NCIH-460 and HEK-293, then incubated for 18 h at 37 °C and 5% CO_2_ prior to treatment with nanoparticles. Nanoparticles (20 µL of PEC-1 to PEC-5, at concentrations of 0.5, 1, and 2 mg/mL) were added into each well and incubated for 68 h at 37 °C and 5% CO_2_. Untreated cells were used as a negative control, while vinblastine was used as a positive control. Cell viability was determined by 3-(4, 5-dimethylthiazol-2-yl)-2,5-diphenyltetrazolium bromide (MTT) assay. Total of 20 µL of MTT in PBS (4 mg/mL) was added to each well and the plates were incubated for a further 4 h. The medium was removed and the precipitated formazan crystals were dissolved in dimethyl sulfoxide (100 µL/well). The absorbance of each well was measured with a PowerWave XS Microplate Reader (λ = 580 nm) (Bio-Tek Instruments Inc.: Vinooski, VT, USA). All experiments were conducted in duplicate.

#### 2.2.7. Dendritic Cells Maturation Assay

Dendritic cells maturation study was performed according to published protocols [34,49]. Naïve Swiss mouse spleen was transferred to a 70-µm cell strainer and gently mashed using a 5 mL syringe plunger. The cell strainer was washed with ACK lysing buffer (0.15 M NH_4_Cl, 1 mM KHCO_3_, 0.1 mM EDTA) and the splenocytes were incubated in lysing buffer for 2–3 min to lyse the erythrocytes. The cells were centrifuged at 350 g for 5 min, then resuspended in RMPI medium supplemented with streptomycin (100 mg/mL), penicillin (100 IU/mL), 2-mercaptoethanol (50 mM), and 10% fetal bovine serum. Cells were plated on a 96-well round bottom plate at a density of 2 × 10^6^ cells/well, then incubated with PBS, MPLA (10 µg/mL), or PEC-1 to PEC-5 (25 µM) at 37 °C for 16 h. The cells incubated with PBS were used as a negative control, while those with MPLA were used as a positive control. The cells were then centrifuged again and the supernatant was removed. Aliquots of 50 µL Fc-block were added to block non-specific binding and cells were further incubated for 10–15 min at 4 °C. The cell suspension was centrifuged again and the supernatant was removed. Cells were then resuspended and incubated for 30 min in the dark at 4 °C with 50 µL of an antibody cocktail containing MHC II (professional APC), CD11c (DC cells), CD80 (late activation), and CD86 (early activation) fluorescent probe-labeled antibodies. After incubation, the cell suspension was centrifuged and resuspend in 100 µL fixation solution, then incubated again for 30 min at 4 °C. Finally, the cells were washed twice with PBS, centrifuged, then resuspend in PBS for further analysis via flow cytometry.

#### 2.2.8. Immunization Study

All animal protocols were approved by The University of Queensland Animal Ethics Committee (SCMB/AIBN/069/17), in accordance with the National Health and Medical Research Council (NHMRC) of Australia (Australian Code of Practice for the Care and Use of Animals for Scientific Purposes, 8th edition 2013). Outbred ARC-Swiss mice (female, 6-weeks old, from The Animal Resource Centre, Perth, Western Australia) were maintained under aseptic conditions with free access to food and water.

Experiment 1: Mice (20 in total) were randomly divided into four groups (*n* = 5). Mice in the negative control group were intranasally administered with PBS (30 µL). Mice in the positive control group were intranasally administered with 30 µg of LCP-1 and 10 µg cholera toxin subunit B (CTB) dissolved in water to a total volume of 30 µL. The other two groups received 30 µL (15 µL per nostril) of either freshly prepared Lip-1 or PEC-1 nanoparticles equivalent to 30 µg of LCP-1 in total. Immunizations were performed on day 0, followed by boosts on days 14 and 28.

Experiment 2: Mice were divided randomly into seven groups, with five mice in each group. The negative and positive control groups were the same as in Experiment 1. The remaining five groups were intranasally administered with freshly prepared PEC nanoparticles (PEC-1–PEC-5). Each mouse received 30 µL of nanoparticles, which was equivalent to 30 µg of LCP-1. The immunization and boost schedule were the same as Experiment 1.

#### 2.2.9. Collection of Saliva and Blood Samples

Saliva samples were collected seven days after the third immunization. Salivation was induced by intraperitoneally injecting mice with 50 μL of pilocarpine solution (0.1%, *w*/*v*). The saliva samples (100 μL from each mouse) were mixed with 2 μL of protease inhibitor (100 mM phenylmethylsulfonylfluoride) in a tube. On day 42, blood samples were collected via cardiac puncture after the mice were euthanized with carbon dioxide. The serum was separated from the samples by centrifugation at 3600 rpm for 10 min. Saliva and sera samples were stored at −80 °C until further analysis.

#### 2.2.10. Measurement of Antibody Titers

J8-specific IgG antibody titers in sera and IgA antibody titers in saliva were measured using enzyme linked immunosorbent assay (ELISA). The measurements were performed following the previously published protocol [46]. Titertek polyvinyl chloride (PVC) 96-well microplates were coated with J8 antigen (0.5 µg/well) in carbonate buffer (pH 9.6) overnight at 4 °C. J8-coated plates were then blocked with 5% (*w*/*v*) skim milk in phosphate-buffered saline for 90 min at 37 °C. Samples were added into the first row of the plate (starting at a 1:100 dilution of sera and 1:4 dilution of saliva), then diluted in a two-fold series down the plate. The plates were incubated for 90 min at 37 °C. The plates were then incubated with secondary antibody (horseradish peroxidase (HRP)-conjugated goat anti-mouse IgG or IgA antibodies) under the same conditions, followed by color development using o-phenylenediamine dihydrochloride (OPD). OPD color develops in proportion to the quantity of antibodies in the sample. The absorbance was measured at 450 nm (SpectraMax microplate reader, Molecular Devices: San Jose, CA, USA). Endpoint titers of IgG and IgA were determined as the highest dilution of samples for which the absorbance was 3 standard deviations (SD) above the mean absorbance of negative control wells [22].

### 2.3. Statistical Analysis

Data were analyzed by one-way analysis of variance (ANOVA), followed by Tukey’s test for comparison of multiple groups with Prism 8.0 (GraphPad Software). *p* values less than 0.05 were considered statistically significant.

## 3. Results and Discussions

### 3.1. Formulation and Characterization

Particle size, shape, and the surface charge of vaccine delivery systems are known to have profound effects on the immunogenicity of loaded antigens [50]. Nanoparticles with a size of 50–200 nm are most effectively taken up and internalized by DCs. Similarly, cationic particles are more easily internalized by APCs because of their electrostatic interactions with anionic APC membranes [29,51]. Therefore, LCP-1 loaded nanoparticles were designed to maintain these advantageous properties.

LCP-1 loaded liposomes were first prepared by thin film hydration. The liposomes were obtained as small, uniform particles following extrusion (144 ± 2 nm, PDI = 0.05). They were positively charged (49 ± 1 mV). Polymer coating of the liposomes relied on electrostatic interactions. The required amount of alginate and TMC was determined by DLS based on the particle size, PDI, and surface charge changes during coating. The end-point of coating was determined as the minimal amount of polyelectrolytes required to produce reverted-charged particles of the smallest size and polydispersity. When positively charged liposomes (containing 100 μg LCP-1) were coated with 30 µg of negatively charged alginate, both the particle size and PDI increased while the surface charge reverted to −21.0 mV. Based on the selected ratio between LCP-1 and alginate, the second layer coating with TMC was optimized following the same procedure as summarized in Supplementary Materials Figure S3a. Lip-1 was produced with an average size of 231 ± 1 nm and a surface charge of 34 ± 2 mV, at the LCP-1:alginate:TMC ratio of 10:6:10 (Table 1).

**Figure 2:**
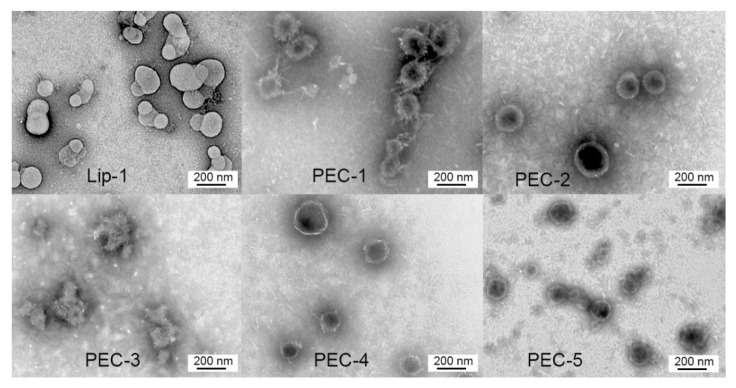
Transmission electron microscope images of LCP-1 loaded nanoparticles, including Lip-1, PEC-1 to PEC-5.

The preparation of PECs was done in two steps: primary complex formation and surface coating with TMC. Both steps were driven by electrostatic interaction between oppositely charged components. In the first step, positively charged LCP-1 and negatively charged polymers were assembled to form primary complexes with the help of sonication. LCP-1 (100 μg) was mixed with different aliquots of negative polymers until uniform, small-sized nanoparticles (<200 nm) with a surface charge of around −30 mV were produced (Supplementary Materials Figure S3). Different quantities of negative polymers were needed for PEC-1–PEC-5 because the total negative charge of each polymer differed (Table 1). In the second step, surface coating with TMC was conducted by adding TMC solution dropwise into the primary complexes to produce the final PEC nanoparticles. An almost identical amount of TMC was required to fully coat all complexes. Both titrations were monitored for particle size and surface charge, as summarized in Supplementary Materials Figure S1. All PECs had similar particle size and surface charge (around 200 nm and + 30 mV, respectively) and size distribution was uniform (Table 1). The morphologies of PECs were measured with TEM and the size of the nanoparticles was consistent with that observed by DLS (Figure 2).

### 3.2. Comparison of the Ability of PEC-1 and Lip-1 to Induce Humoral Immune Responses

We investigated the induction of humoral responses in mice by intranasal immunization with LCP-1 delivered with either polymer coated liposomes or PEC nanoparticles. Liposomes hold great potential for intranasal vaccine delivery because of their adjuvanting potency [22]. Furthermore, coating the liposomes with polymer can enhance stability, protect the encapsulated cargo from leakage [24], and reduce cytotoxicity caused by high positive surface charge [52]. We previously prepared alginate and TMC coated liposomes for oral vaccine delivery against GAS in order to improve the stability of liposomes in gastroenteric conditions and enhance muco-adhesion and membrane permeation at mucosal sites [23]. These orally delivered polymer-coated liposomes stimulated higher serum IgG and mucosal IgA antibody titers in outbred mice compared to liposomes. When liposomes were coated with several layers of polymers, the liposomal surface was not exposed to the physiological environment. Therefore, we hypothesized that lipid bilayers can be completely omitted in formulations, and simplified liposome-free PEC nanoparticles can be produced and examined as vaccine delivery systems.

**Figure 3:**
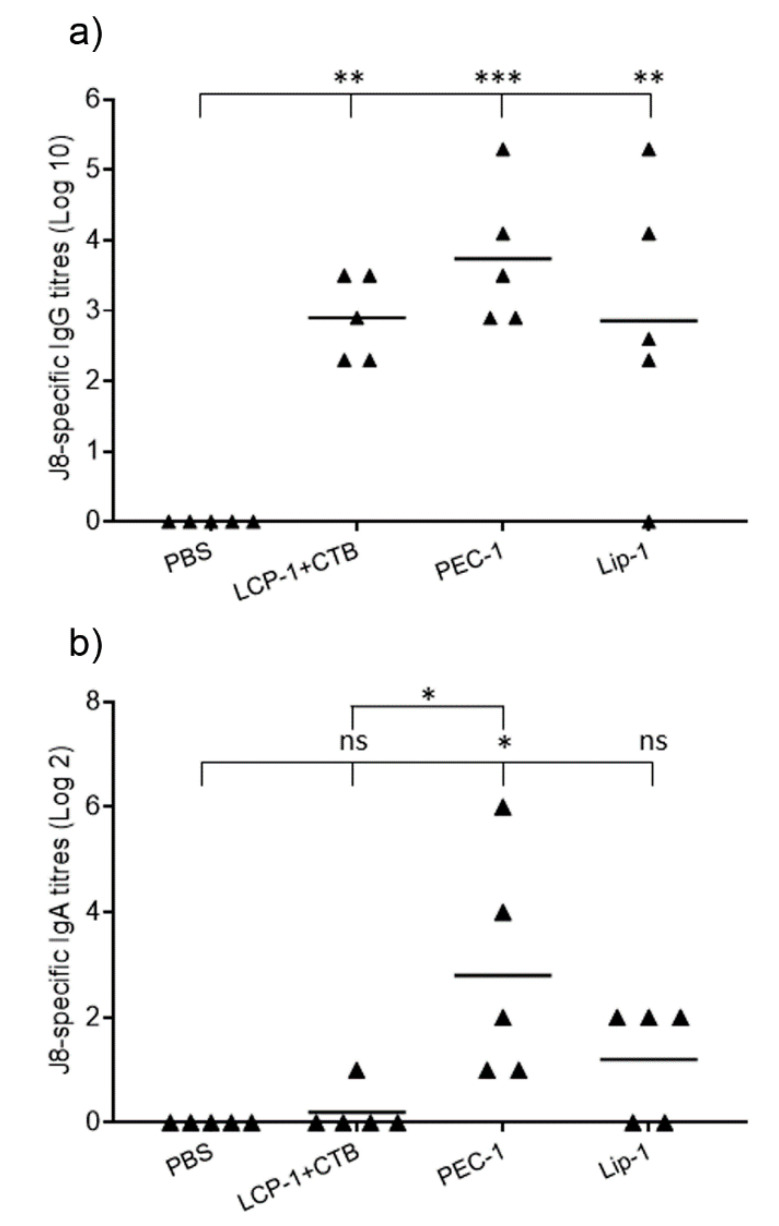
J8-specific serum IgG (**a**) and salivary IgA (**b**) titers in outbred Swiss mice models after second boost (experiment 1). ns, *p* > 0.05; * *p* < 0.05; ** *p* < 0.01; *** *p* < 0.001. PBS: phosphate buffer solution, CTB: cholera toxin B.

The ability of LCP-1-loaded alginate/TMC-coated liposome (Lip-1) and LCP-1/alginate/TMC PEC nanoparticles (PEC-1) to induce humoral immune responses following intranasal immunization was examined in mice. Both nanoparticles and positive control solutions (LCP-1 adjuvanted with classical mucosal adjuvant CTB) induced significantly higher serum IgG titers compared to PBS (Figure 3a). PEC-1 induced higher IgG titers than Lip-1; however, the difference was not statistically significant. Analysis of salivary IgA titers also revealed a similar trend in mucosal immune responses (Figure 3b). However, only PEC-1 induced significantly higher IgA titers compared to PBS (*p* < 0.05). Encapsulation of LCP-1 into liposomes prior to polymer coating (Lip-1) did not induce stronger systemic or mucosal immune responses in outbred mice in comparison to LCP-1-loaded PECs (PEC-1). The similar levels of humoral immune responses between Lip-1 and PEC-1 resulted from their similar outer-surface characteristics, such as particle size, surface charge, and the same coating materials (e.g., alginate and TMC). Therefore, PEC nanoparticles were selected for further optimization. Hence, the known shortcomings of liposomes in vaccines, such as the requirement for organic solvent and complicated and redundant preparation procedures, could be omitted.

### 3.3. Comparison of PECs’ Ability to Induce Systemic and Humoral Immune Responses

**Figure 4:**
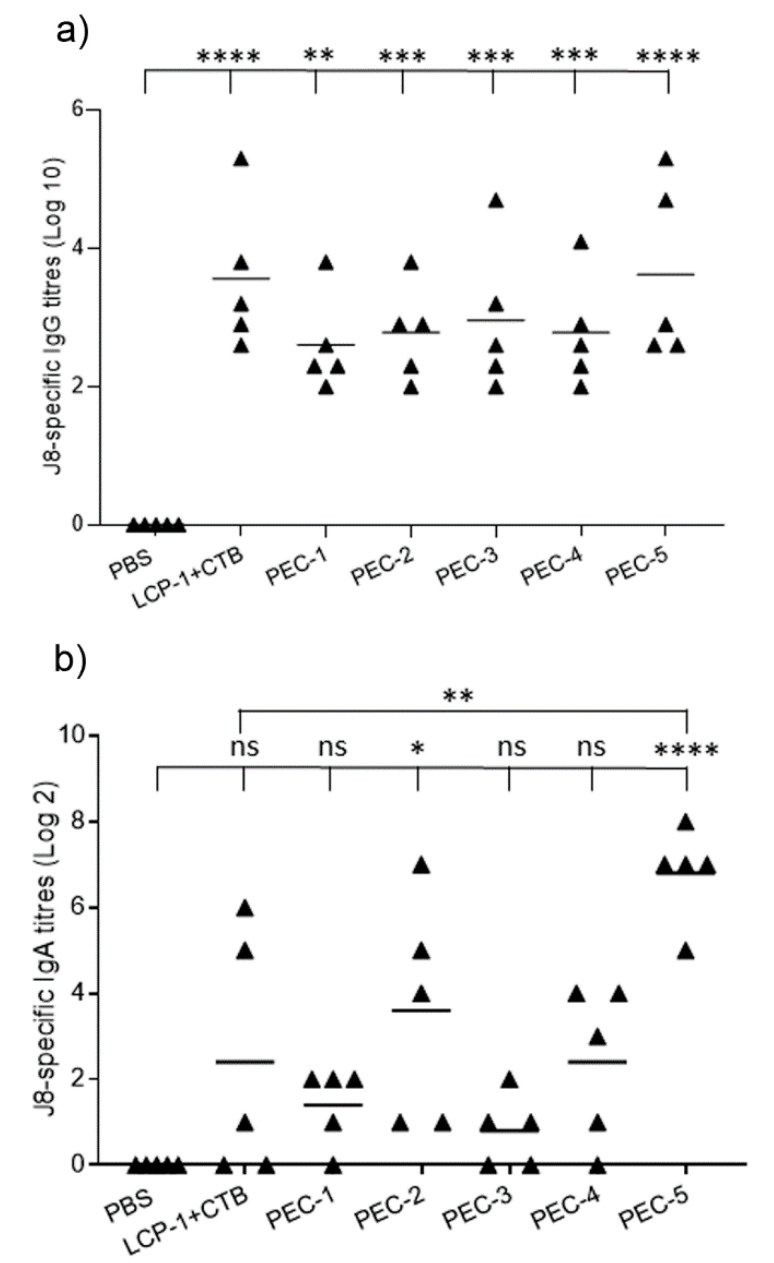
J8-specific serum IgG (**a**) and salivary IgA (**b**) titers in outbred Swiss mice models after second boost (experiment 2). ns, *p* > 0.05; * *p* < 0.05; ** *p* < 0.01; *** *p* < 0.001; **** *p* < 0.0001. PBS: phosphate buffer solution, CTB: cholera toxin B.

LCP-1-loaded PEC nanoparticles composed of different negative polymers and coated with TMC were tested for their ability to induce systemic and mucosal immune responses in outbred mice. TMC has been extensively studied in vaccine delivery systems because of its adjuvanting activity; however, the impact of interbedded negative polymers has not been examined. Therefore, we investigated the influence of the negative polymers within PECs in terms of the immunogenicity of LCP-1 in outbred Swiss mice. A series of PECs were developed by mixing positively charged LCP-1 and different negatively charged polymers followed by TMC coating. In such three-component PEC systems, negative polymers, besides serving as a complexing agent between antigen and TMC, may play an important role in enhancing immune responses against GAS. To test this, mice were immunized with LCP-1 (30 µg) either in the form of a physical mixture with CTB or loaded into PEC-1 to PEC-5. Following the second boost, J8-specific serum IgG and salivary mucosal IgA antibody titers were examined (Figure 4). All immunized mice produced higher serum IgG titers compared to the negative control group (PBS). Considering the same surface coating material (TMC) at the outermost layer, and the similar particle size and surface charge of LCP-1 loaded PECs, the different levels of induced antibody titers resulted from the presence of different negative polymers in the PECs. Among the five LCP-1-loaded PECs, heparin-bearing PEC-5 elicited higher antibody (IgG) titers than the other vaccine candidates, though the difference was not statistically significant.

A vaccine that can induce mucosal immune responses against GAS would not only prevent systemic invasion of the bacterium but also eliminate local throat colonization by GAS, and therefore block disease carriers to stop the transmission of infection. Thus, the induction of mucosal immunity and the production of secretory IgA, the major mucosal immunoglobulin, are highly desirable [53,54]. Outbred Swiss mice immunized with PEC-5 generated significantly higher IgA antibody titers than the negative control group (*p* < 0.0001) and the other experimental groups, except for PEC-2 (Figure 4). PEC-2 also induced higher IgA titers compared to PBS (*p* < 0.05) in spite of the overall low IgA responses. The superiority of heparin in PEC-5 might be a result of the high proportion of sulfate groups on its repeating monosaccharide units, which can interact more strongly with TMC amino groups than carboxy groups. It has also been suggested that sulfated polymers have stronger biological activities, including adjuvanticity [55]. However, it has to be taken into account that dextran and chondroitin sulfate also carry sulfate groups. In addition, heparin itself has been reported as being able to stimulate cellular immune responses and potentially enhance humoral immune responses [56,57,58].

Considering both systemic and mucosal immune responses, PEC-5 was the most immunogenic complex following intranasal immunization in outbred Swiss mice. The immune-stimulating activity of PEC-5 was also verified in an in vitro DC maturation study (Supplementary Materials Figure S4). DCs are key factors in innate and adaptive immune responses. Immature DCs take up antigens, then convert into mature DCs, which process and present antigens to T-cells and subsequently trigger antigen-specific immune responses [59]. Mature DCs overexpress a series of cell surface markers, such as CD 86, CD 80, and MHC-II, which are responsible for antigen presentation to CD4 + T-cells. After 5 h incubation with PBS (negative control), MPLA (positive control), and PEC-5, DCs exhibited no activation under any condition. Treatment with PEC-5 for 16 h significantly upregulated the expression of CD 80 (*p* < 0.05, compared with PBS), while the expression of CD 86 and MHC II increased but without statistical significance, indicating that PEC-5 was effective in activating DCs and most DCs were in the late stage of maturation after 16 h incubation.

Biocompatibility is an important consideration for vaccine/drug delivery systems. The high surface charge of nanoparticles is considered to be a key factor in cytotoxicity, as the electrostatic interactions between positively charged nanoparticles and negatively charged cell membranes could disturb and even rupture cell membranes [60]. The developed antigen-loaded PECs (PEC-1–PEC-5) had a surface charge of around +30 mv and, therefore, were expected to be safe for biological applications. All of the PECs were also prepared with naturally derived polymers, which were biocompatible and biodegradable. Thus, a lack of cytotoxicity was anticipated. To verify this, the cytotoxicity of PEC-5 was examined at three different concentrations (0.5, 1, 2 mg/mL) in SW620, NCIH-460, and HEK-293 cell lines. PEC-5 was not cytotoxic against any of the three cell lines, with more than 50% of cells still observed to be viable after 68 h of incubation at 37 °C (Supplementary Materials Figure S5). In addition, cell viability was not concentration-dependent, as it showed no evident relationship with increasing PEC concentration.

## 4. Conclusions

In this study, PECs and polymer coated liposomes were constructed and examined for the delivery of nasal vaccines against GAS. LCP-1 loaded nanoparticles with similar size and surface charge were produced and tested as vaccine candidates against GAS in outbred mice. PEC-1 induced stronger humoral immune responses than alginate/TMC coated liposomes (Lip-1), indicating that an inner liposomal bilayer was not necessary for enhancing the immunogenicity of antigens. Thus, liposome-free PEC nanoparticles were selected for further optimization. These nanoparticles were easier to prepare through an organic solvent-free process and were more cost-effective. PECs composed of various negative polymers (alginate, chondroitin sulfate, dextran, hyaluronic acid, and heparin) and coated with TMC were fabricated and evaluated for their ability to induce antigen-specific immune responses. PEC-5 composed of LCP-1, heparin, and TMC induced the strongest humoral immune responses in outbred mice, in comparison with other PECs. Therefore, we found that negative polymers assisted in eliciting immune responses in addition to functioning as complexing agents. Moreover, PECs were produced with biocompatible and biodegradable polymers, so were safe for biological purposes. Owing to the mild preparation conditions and variety in biocompatible polymers, the PEC system may be readily adopted for the delivery of other peptide-based vaccines and drugs. This work provides new insight into vaccine delivery/adjuvanting strategies and, for the first time, examined the impact of different negative polymers in PECs. The mechanism of heparin adjuvant activity in the PECs warrants further investigation.

## Figures and Tables

**Table 1:** Physicochemical characterization of LCP-1 loaded nanoparticles.

Formulation	Negative Polymer	Positive Polymer	Particle Size (nm)	PDI	Zeta Potential (mV)	Mass Ratio *
Lip-1	alginate	TMC	231 ± 1	0.176 ± 0.02	33.8 ± 1.7	10:6:10
PEC-1	alginate	TMC	237 ± 4	0.199 ± 0.01	29.3 ± 1.5	10:4:8
PEC-2	chondroitin sulfate	TMC	227 ± 2	0.133 ± 0.01	27.2 ± 0.2	10:5:10
PEC-3	dextran	TMC	182 ± 1	0.193 ± 0.01	31.9 ± 0.6	10:2.5:8
PEC-4	hyaluronic acid	TMC	199 ± 4	0.235 ± 0.01	29.6 ± 0.5	10:8:8
PEC-5	heparin	TMC	206 ± 4	0.177 ± 0.01	29.5 ± 0.4	10:3:8

PDI: polydispersity index, TMC: trimethyl chitosan. * Mass ratio = LCP-1: negative polymer: TMC.

## Supplementary Materials

The following are available online at https://www.mdpi.com/2079-4991/10/5/823/s1. Figure S1: Analytical HPLC profile of LCP-1. t_R_ = 23.0 min. Figure S2. Mass spectrum of LCP-1. ESI-MS: m/z 1203.8 (calculated 1203.8) [M + 5H]^5+^; 1003.1 (calculated 1003.4) [M + 6H]^6+^; 860.0 (calculated 860.2) [M + 7H]^7+^; 752.8 (calculated 752.8) [M + 8H]^8+^; 669.2 (calculated 669.2) [M + 9H]^9+^. Figure S3. The optimization of formulation monitored with DLS. In each panel, the top two graphs represented the optimization of negative polymer mixing ratios (A, Lip-1; B, PEC-1; C, PEC-2; D, PEC-3; E, PEC-4; F, PEC-5) while the bottom two graphs represented the optimization of TMC coating. The optimum amount of each polymer required was marked in blue column. Figure S4. PEC-5 induced maturation of splenocyte-derived DCs. DCs were cultured with PEC-5 for 5 h and 16 h separately. Expression levels of CD 86 (a), CD 80 (b), and MHC-II (c) were measured by flow cytometry. Results are mean fluorescence intensity (MFI) ± SEM (*n* = 2). * *p* < 0.05, ** *p* < 0.01 and *** *p* < 0.001. Figure S5. The viability of cells treated with PEC-5. NCIH-460: human lung cancer cell line, SW620: human colorectal cancer cell line, HEK-293: human kidney cell line. After 68 h incubation, cell viability was measured with MTT assay. All values are reported as means ± SEM with duplicate data points.
