# Supplementary material for: Development of Polyelectrolyte Complexes for the Delivery of Peptide-Based Subunit Vaccines against Group A Streptococcus
Source: Nanomaterials (Basel). 2020 Apr 26;10(5):823. doi: 10.3390/nano10050823 (PMC7712447; doi:10.3390/nano10050823)
Supplement: Supplementary file 1 [file nanomaterials-10-00823-s001.pdf]

Supporting information

Development of polyelectrolyte complexes for the delivery of peptide-based subunit vaccines against group A streptococcus

Lili Zhao [1], Wanli Jin [1], Jazmina Gonzalez Cruz [2], Nirmal Marasini [1], Zeinab G. Khalil [3], Robert J. Capon [3], Waleed M. Hussein [1,4], Mariusz Skwarczynski [1]* and Istvan Toth [1,3,5]*

[1] School of Chemistry & Molecular Biosciences, The University of Queensland, St Lucia, QLD 4072, Australia; lili.zhao@uq.edu.au (L.Z.); wanli.jin@uq.net.au (W.J.); nirmal.marasini@uq.net.au (N.M.); w.hussein@uq.edu.au (W.M.H.)

[2] Diamantina Institute, Translational Research Institute, The University of Queensland, Wooloongabba, QLD 4102, Australia; j.gonzalezcruz@uq.edu.au (J.G.C.)

[3] Institute for Molecular Bioscience, The University of Queensland, St. Lucia, QLD 4072, Australia; z.khalil@imb.uq.edu.au (Z.G.K.); r.capon@imb.uq.edu.au (R.J.C.)

[4] Pharmaceutical Organic Chemistry Department, Faculty of Pharmacy, Helwan University, Helwan, 11795, Egypt

[5] School of Pharmacy, The University of Queensland, Woolloongabba, QLD 4102, Australia

* Correspondence: m.skwarczynski@uq.edu.au (M.S.); i.toth@uq.edu.au (I.T.)

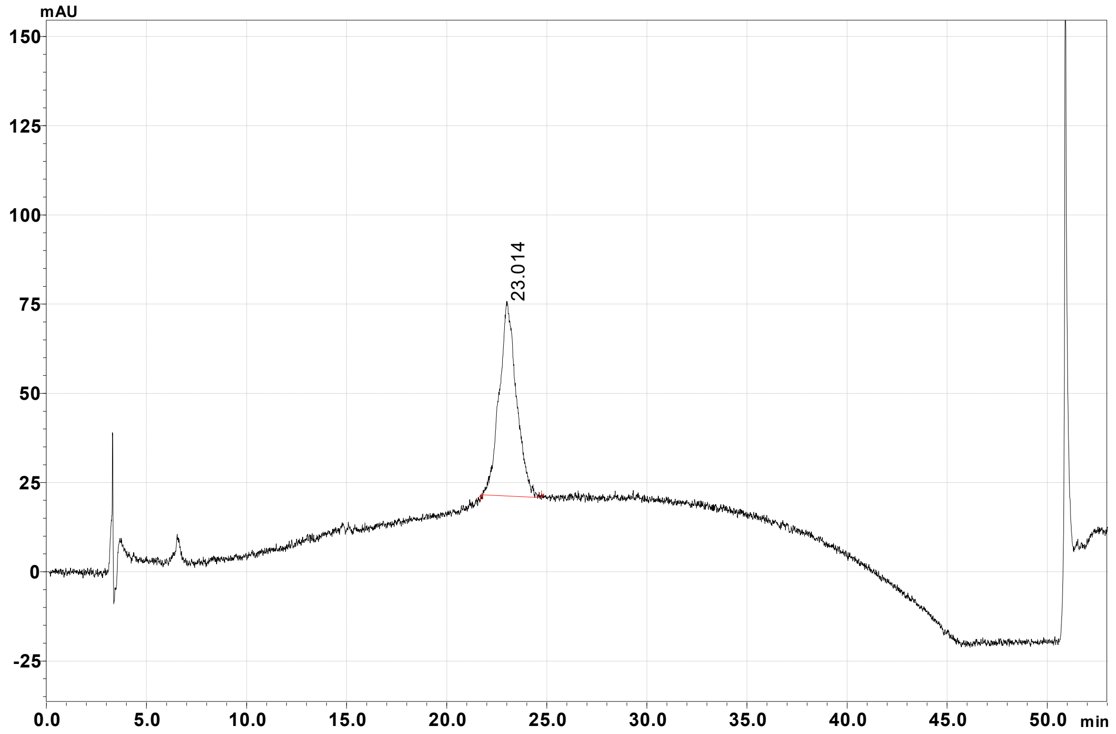

Figure S1. Analytical HPLC profile of LCP-1. t_R=23.0 min

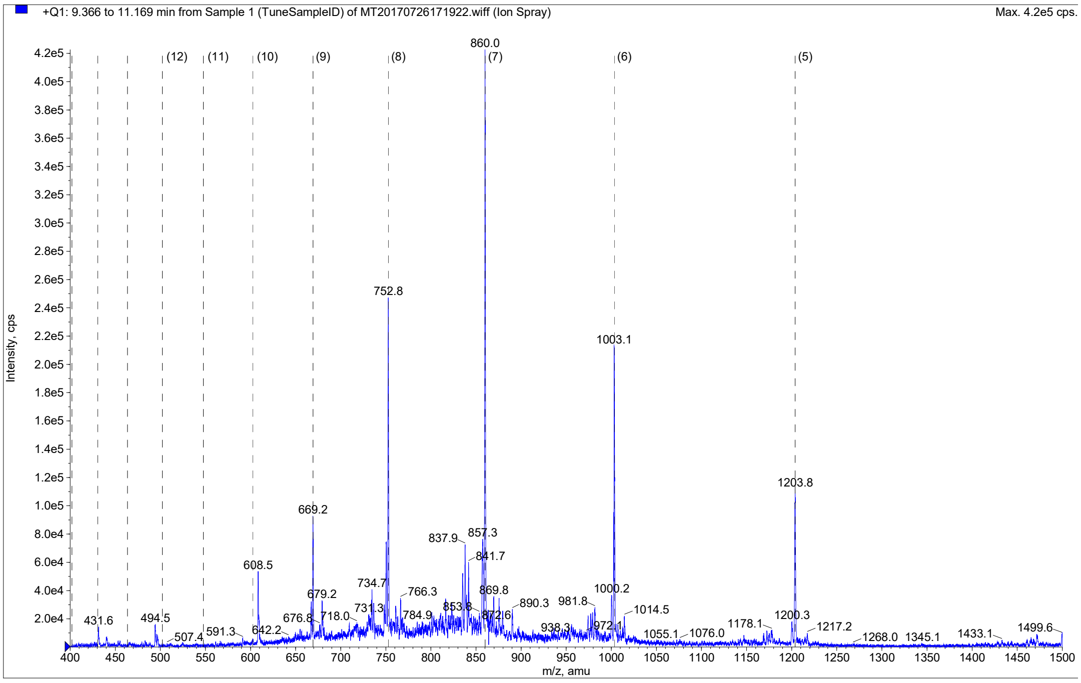

Figure S2. Mass spectrum of LCP-1. ESI-MS: m/z 1203.8 (calculated 1203.8) $[M+5H]^{5+}$; 1003.1 (calculated 1003.4) $[M+6H]^{6+}$; 860.0 (calculated 860.2) $[M+7H]^{7+}$; 752.8 (calculated 752.8) $[M+8H]^{8+}$; 669.2 (calculated 669.2) $[M+9H]^{9+}$

a)

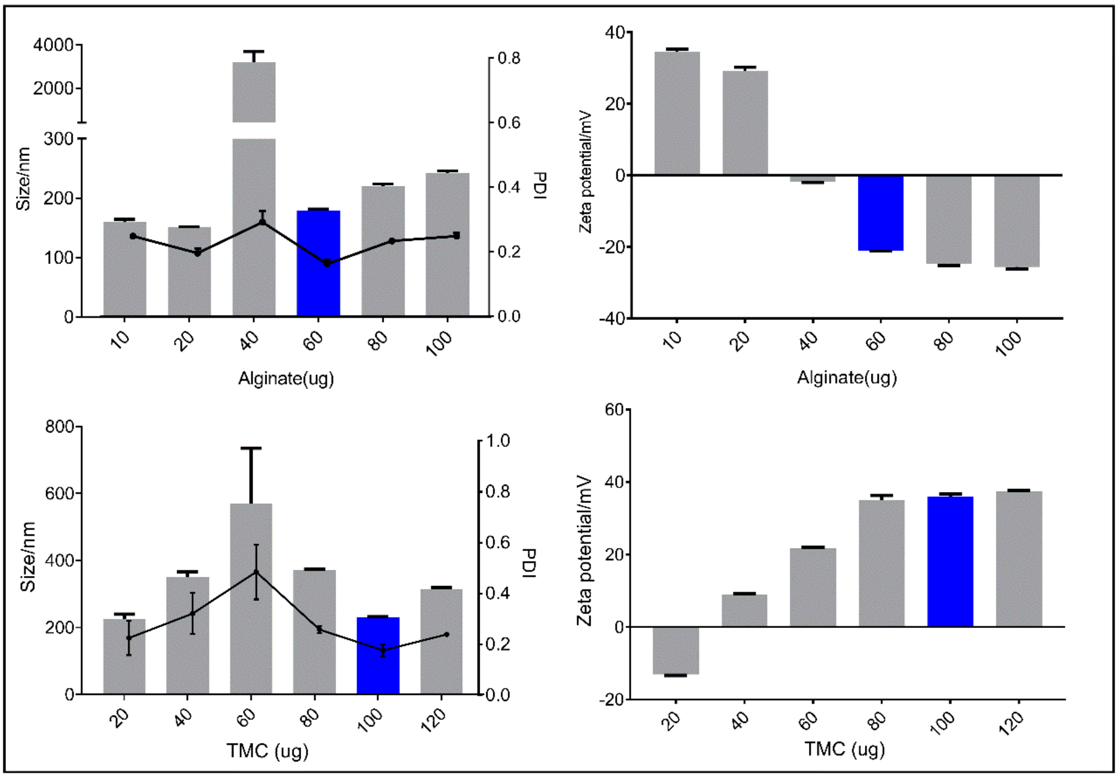

b)

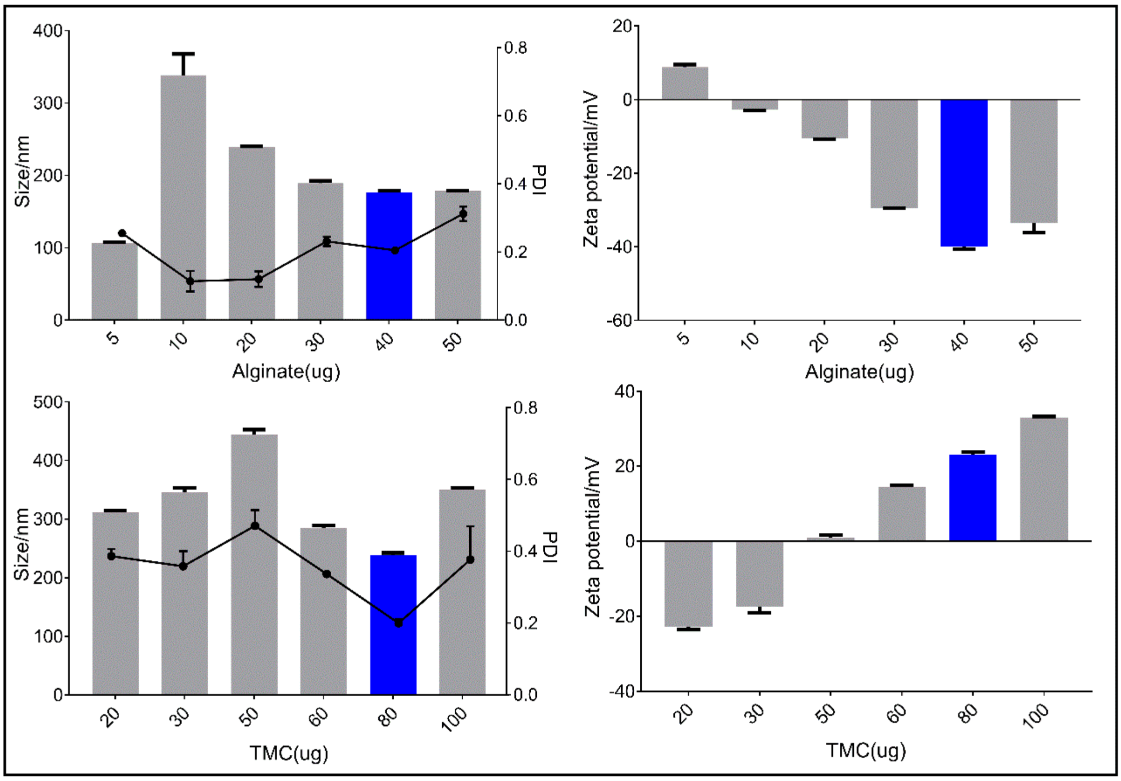

Size/nm
PDI
Alginate(ug)
Zeta potential/mV
Alginate(ug)
Size/nm
PDI
TMC(ug)
Zeta potential/mV
TMC(ug)

c)

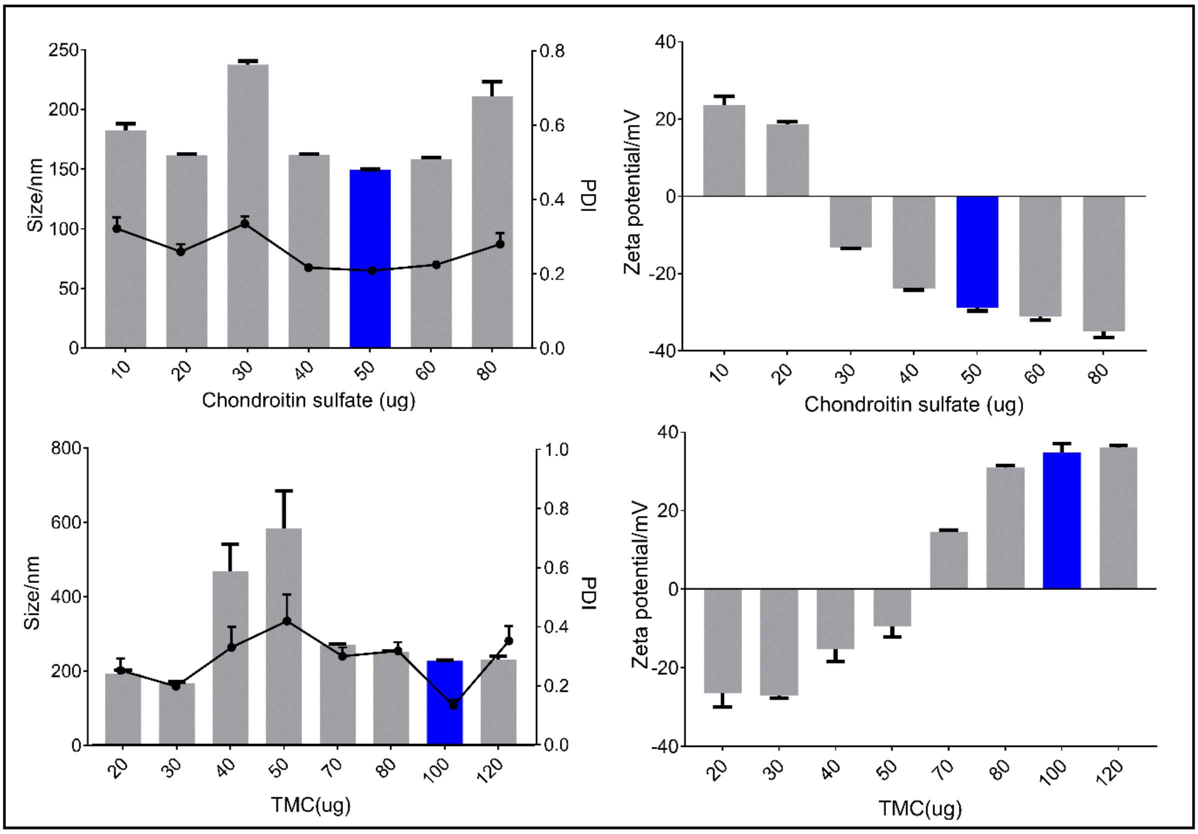

Size/nm
PDI
Chondroitin sulfate (ug)
Zeta potential/mV
Chondroitin sulfate (ug)
Size/nm
PDI
TMC(ug)
Zeta potential/mV
TMC(ug)

d)

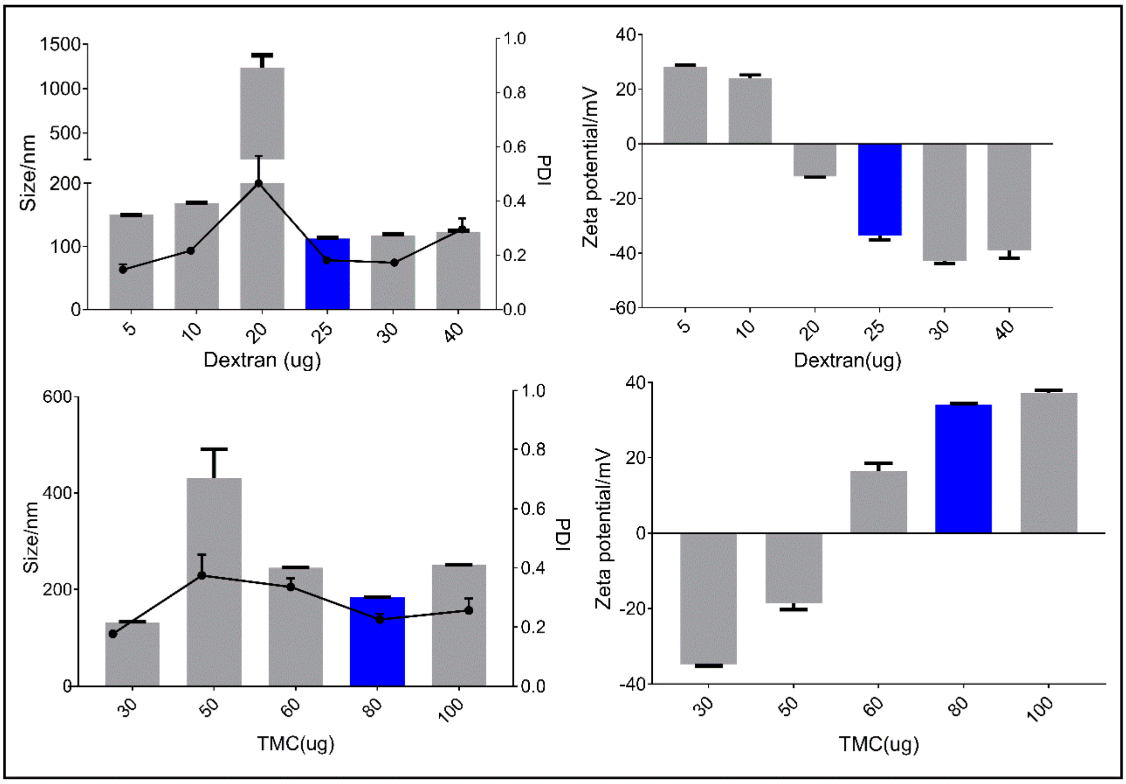

Size/nm
PDI
Dextran (ug)
Zeta potential/mV
Dextran(ug)
Size/nm
PDI
TMC(ug)
Zeta potential/mV
TMC(ug)

e)

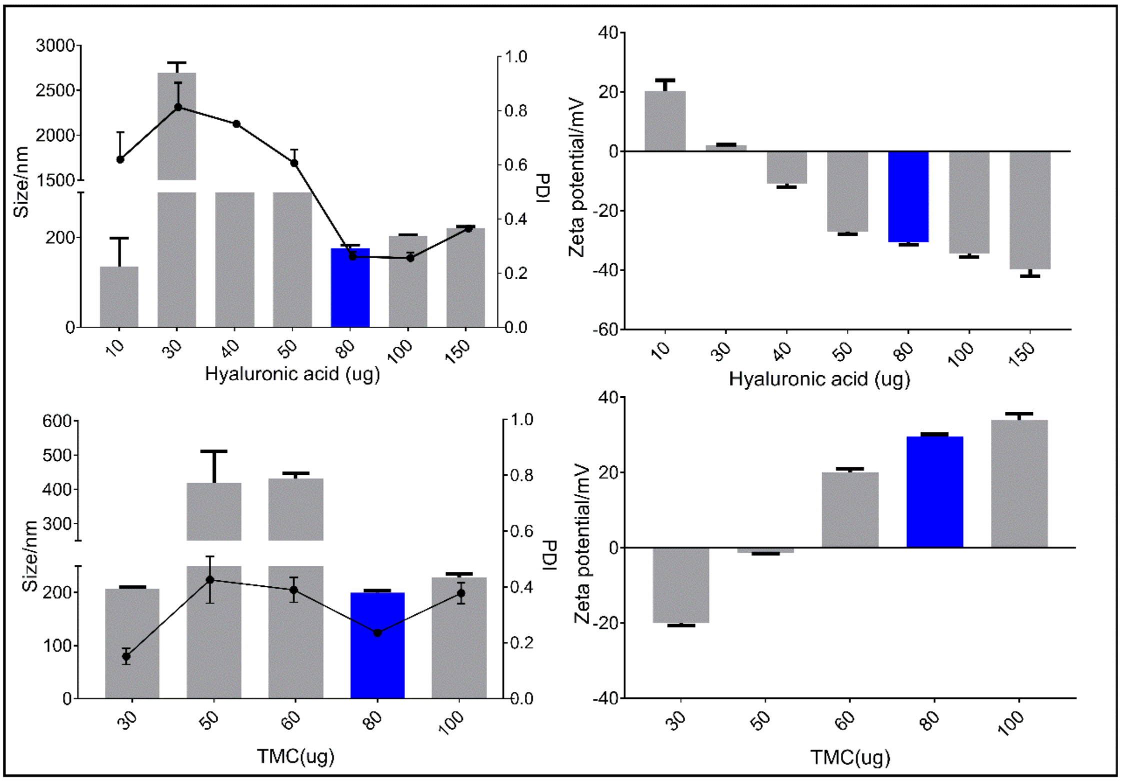

Size/nm
PDI
Hyaluronic acid (ug)
Zeta potential/mV
Hyaluronic acid (ug)
Size/nm
PDI
TMC(ug)
Zeta potential/mV
TMC(ug)

f)

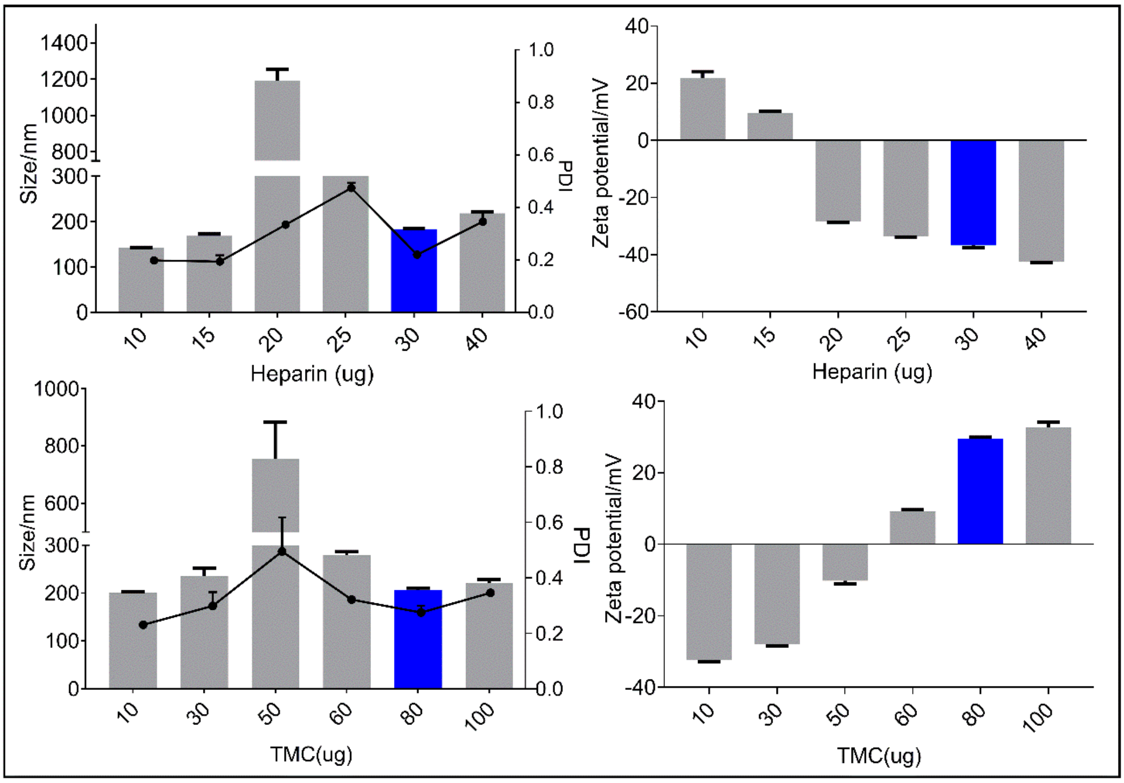

Figure S3. The optimization of formulation monitored with DLS. In each panel, the top two graphs represented the optimization of negative polymer mixing ratios (a, Lip-1; b, PEC-1; c, PEC-2; d, PEC-3; e, PEC-4; f, PEC-5) while the bottom two graphs represented the optimization of TMC coating. The optimum amount of each polymer required was marked in blue column.

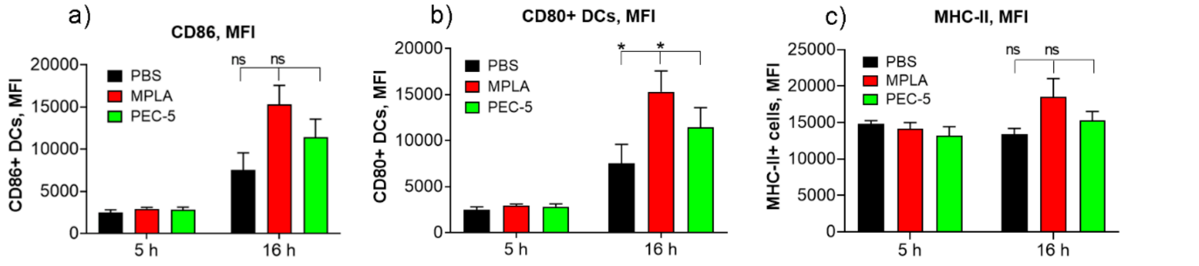

Figure S4. PEC-5 induced maturation of splenocyte-derived DCs. DCs were cultured with PEC-5 for 5 h and 16 h separately. Expression levels of CD 86 (a), CD 80 (b) and MHC-II (c) were measured by flow cytometry. Results are mean fluorescence intensity (MFI) ± SEM (n= 2). * $p<0.05$, ** $p<0.01$ and *** $p<0.001$.

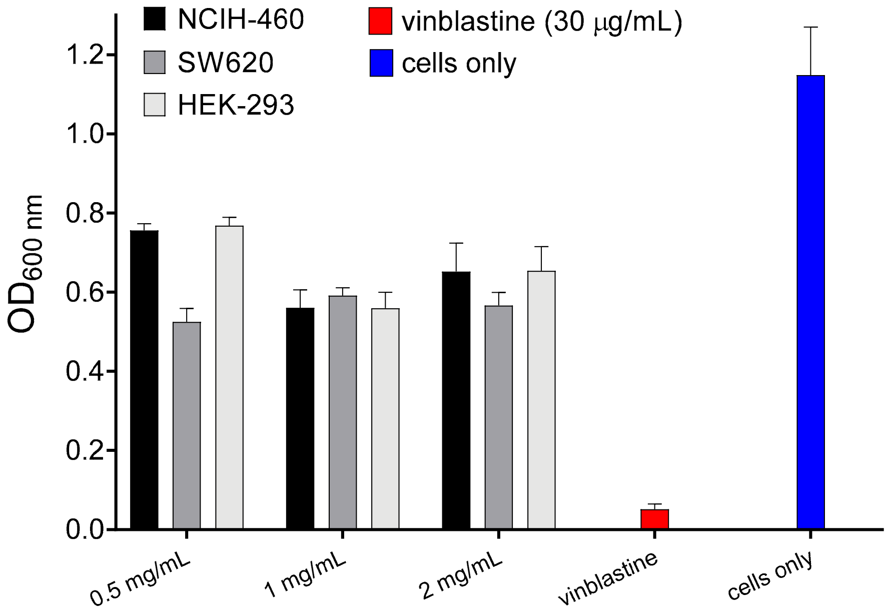

Figure S5. The viability of cells treated with PEC-5. NCIH-460: human lung cancer cell line, SW620: human colorectal cancer cell line, HEK293: human kidney cell line. After 68 h incubation, cell viability was measured with MTT assay. All values are reported as means ± SEM with duplicate data points.
